# High-Efficiency Deep-Blue Solution-Processed OLED Devices Enabled by New Dopant Materials

**DOI:** 10.3390/ma18102213

**Published:** 2025-05-10

**Authors:** Saeyoung Oh, Hyukmin Kwon, Sangwook Park, Seokwoo Kang, Sang-Tae Kim, Kiho Lee, Hayoon Lee, Jongwook Park

**Affiliations:** Integrated Engineering, Department of Chemical Engineering, Kyung Hee University, Yongin 17104, Gyeonggi, Republic of Korea; tpdud5821@khu.ac.kr (S.O.); hm531@khu.ac.kr (H.K.); pswook@khu.ac.kr (S.P.); swkang@khu.ac.kr (S.K.); kimst2@khu.ac.kr (S.-T.K.); kiholee@khu.ac.kr (K.L.); kssarang1@khu.ac.kr (H.L.)

**Keywords:** OLED, solution process, dopant, TDBA, carbazole

## Abstract

Two blue fluorescent dopants were designed and successfully synthesized, 5-(2,12-di-tert-butyl-5,9-dioxa-13b-boranaphtho [3,2,1-de]anthracen-7-yl)-5H-benzo[b]carbazole (TDBA-Bz) and 9-(2,12-di-tert-butyl-5,9-dioxa-13b-boranaphtho[3,2,1-de]anthracen-7-yl)-9H-carbazole (TDBA-Cz). Both in solution and the film state, the two emitters demonstrated deep-blue luminescence characteristics. In solution-processed organic light-emitting diodes (OLEDs), TDBA-Bz and TDBA-Cz used as dopant materials showed electroluminescence peaks at 436 nm and 413 nm, respectively. The corresponding CIE color coordinates were determined to be (0.181, 0.114) for TDBA-Bz and (0.167, 0.086) for TDBA-Cz. The solution-processed device using TDBA-Cz as a dopant exhibited a current efficiency (CE) of 7.25 cd/A and an external quantum efficiency (EQE) of 6.45%, demonstrating higher efficiencies compared to the device with TDBA-Bz. In particular, at a luminance of 2000 cd/m^2^, TDBA-Cz maintained an EQE of 4.81%, with only a slight decrease from its maximum EQE.

## 1. Introduction

Organic light-emitting diodes (OLEDs), which have garnered significant attention across various fields, rely heavily on the performance of the emitting layer (EML), a key component that directly affects device efficiency and operational stability [1,2,3,4,5]. However, achieving highly efficient and color-pure blue emitters remains a significant technological challenge. To date, most reported deep-blue OLEDs have been fabricated using vacuum deposition processes to ensure high efficiency and long device lifetimes [6,7,8,9,10]. Currently, the widely used vacuum deposition process is primarily applied to smartphone displays utilizing GEN 5 mother glass. Although large-area displays such as TVs have been commercialized, they are still limited by the need to use an open-mask vacuum deposition method. Therefore, continuous efforts are being made to replace the costly vacuum deposition process with solution-based processes for large-area displays, including TVs, desktop monitors, and automotive displays. Accordingly, there is a need for the development and research of various new materials suitable for solution processing. Although vacuum deposition allows for the precise fabrication of multilayer structures, it is associated with high processing costs and limited scalability due to the use of expensive vacuum equipment, low material utilization, and difficulties in applying the process to large-area substrates. Therefore, advancing solution-processable small-molecule organic emitters that are compatible with spin-coating and inkjet printing techniques is critical for the commercialization of next-generation OLED displays. Solution-processable small-molecule emitters offer advantages over polymeric materials in terms of solubility and ease of purification, and their molecular structures can be finely tuned to achieve both high emission efficiency and stability. These features contribute to process simplification and cost-effective manufacturing. Consequently, solution-processable blue dopants are emerging as a pivotal technology in developing next-generation OLEDs [11,12,13,14,15].

In this study, we designed a molecule aimed at achieving high-efficiency blue emission in solution-processed OLED devices. 5,9-Dioxa-13b-boranaphtho[3,2,1-de]anthracene (DOBNA) was initially chosen as the core structure to improve structural stability and reduce non-radiative decay pathways [16,17]. Planar moieties such as DOBNA can suppress intramolecular vibrations and rotations, thereby reducing energy loss during the emission process and enabling deep-blue emission with superior color purity characterized by a narrow full width at half maximum (FWHM). Second, to improve the carrier balance between holes and electrons, a bipolar molecular structure was designed. Since DOBNA exhibits electron-transporting characteristics, the introduction of electron-donating units such as carbazole (Cz) and benzocarbazole (Bz) enables balanced hole and electron injection and transport. This facilitates effective confinement of the exciton recombination zone within the EML, thereby ensuring stable emission characteristics even under high-brightness operating conditions. This strategy also enables excellent film quality and charge transport properties in solution-processed devices, contributing effectively to the suppression of severe efficiency roll-off under high-brightness operation [18,19,20,21,22]. Third, steric hindrance was introduced between the DOBNA core and the electron-donating units. While materials used in solution processing require good solubility, the highly planar nature of DOBNA leads to strong intermolecular interactions. In such cases, the strong intermolecular interactions can significantly reduce solubility, leading to the material being unsuitable for solution-based processing. Therefore, the incorporation of moieties such as Cz and Bz, which can hinder molecular packing, is essential. By carefully selecting the substitution positions, the dihedral angle between the DOBNA and the donor core can be tuned, thereby enhancing solubility.

Recently, a number of studies with similar approaches have been reported. J. Woo et al. carried out a comprehensive analysis of various RGB OLED devices fabricated via solution processing and reported that the use of small-molecule emitters offers advantages over polymer-based systems in terms of solution orthogonality. This allows for effective suppression of interlayer dissolution, enabling the fabrication of more precise and stable OLED devices [23]. J. Hwang et al. developed a series of dopants by introducing carbazole-derived groups to TDBA, a DOBNA-based backbone substituted with tert-butyl groups. Among them, BO-tCzPhICz achieved high solubility for solution processing by utilizing the twisted dihedral angle between the donor and acceptor units. When applied as a dopant in a solution-processed electroluminescent (EL) device, the material exhibited excellent performance, with a maximum external quantum efficiency (EQE_max_) of 17.8%, a maximum electroluminescence (EL_max_) at 436 nm, an FWHM of 56 nm, and CIE coordinates of (0.15, 0.077) [24]. K. Jiang et al. designed ICz-BO by combining a TDBA acceptor with an indolocarbazole-derived donor. When employed as a dopant in a solution-processed EL device, the material achieved an EQE_max_ of 12.0%, with an EL_max_ at 414 nm, a narrow FWHM of 37 nm, and CIE coordinates of (0.164, 0.031), demonstrating violet-blue emission characteristics [25]. In this study, two dopants—5-(2,12-di-tert-butyl-5,9-dioxa-13b-boranaphtho[3,2,1-de]anthracen-7-yl)-5H-benzo[b]carbazole (TDBA-Bz) and 9-(2,12-di-tert-butyl-5,9-dioxa-13b-boranaphtho[3,2,1-de]anthracen-7-yl)-9H-carbazole (TDBA-Cz)—were designed and synthesized. Both Cz and Bz are well known as structurally stable donors with strong electron-donating effects and are expected to enhance the hole-transporting properties of OLED devices [26,27,28,29]. Accordingly, investigations into the optical, thermal, and electroluminescent behaviors of the two newly synthesized bipolar materials were conducted, focusing on differences in the donor units.

## 2. Materials and Methods

### 2.1. Synthesis and Characterization

#### 2.1.1. Synthesis of 4,4′-((2,5-Dibromo-1,3-phenylene)bis(oxy))bis(tert-butylbenzene) (**1**)

A mixture of 2,5-dibromo-1,3-difluorobenzene (10.0 g, 36.8 mmol), 4-tert-butylphenol (16.6 g, 110 mmol), and potassium carbonate (K_2_CO_3_) (20.3 g, 147 mmol) was dissolved in 120 mL of dimethylformamide (DMF) under N_2_ conditions. The reaction was stirred at 150 °C for 20 h. Following cooling to room temperature, the reaction mixture was quenched in water and extracted repeatedly with dichloromethane. The collected organic extracts were dried using anhydrous MgSO_4_, evaporated under reduced pressure, and further purified via silica gel column chromatography (dichloromethane (MC):n-hexane = 1:9, *v*/*v*), providing a white solid in 71% yield.^1^H NMR (400 MHz, Chloroform-d) δ 7.40–7.38 (d, J = 8.7 Hz, 4H), 6.98–6.96 (d, J = 8.8 Hz, 4H), 6.72 (s, 2H), 1.33 (s, 18H).

#### 2.1.2. Synthesis of 7-Bromo-2,12-di-tert-butyl-5,9-dioxa-13b-boranaphtho[3,2,1-de]anthracene (**2**)

Under N_2_ conditions, Compound **1** (3.00 g, 5.64 mmol) was dissolved in anhydrous m-xylene (30 mL). After stirring for 15 min, a solution of n-butyllithium (n-BuLi, 2.48 mL, 2.5 M in hexane, 6.20 mmol) was added dropwise at −50 °C, and the reaction was continued at room temperature for 1 h. Subsequently, the mixture was cooled to −30 °C, and boron tribromide (0.5 mL, 5.32 mmol) was added slowly. The solution was then heated to 40 °C and stirred for 1.5 h. At 0 °C, N,N-diisopropylethylamine (DIPEA, 1.54 mL, 8.87 mmol) was introduced, followed by stirring for 30 min at room temperature. The mixture was heated at 140 °C for 20 h to complete the reaction. Following cooling to room temperature, the reaction mixture was quenched in water and extracted repeatedly with dichloromethane. The collected organic extracts were dried using anhydrous MgSO_4_, evaporated under reduced pressure, and further purified via silica gel column chromatography (MC:n-hexane = 1:4, *v*/*v*), providing a white solid in 38% yield. ^1^H NMR (400 MHz, Chloroform-d) δ 8.73–8.72 (d, *J* = 2.5 Hz, 2H), 7.79–7.77 (m, *J* = 8.8 Hz, 2H), 7.48–7.46 (d, *J* = 8.8 Hz, 2H), 7.37 (s, 2H), 1.47 (s, 18H).

#### 2.1.3. Synthesis of 5-(2,12-Di-tert-butyl-5,9-dioxa-13b-boranaphtho[3,2,1-de]anthracen-7-yl)-5H-benzo[b]carbazole (TDBA-Bz) (**3**)

Under N_2_ conditions, Compound **2** (1.00 g, 2.17 mmol), 5H-benzo[b]carbazole (0.57 g, 0.26 mmol), and sodium tert-butoxide (NaOtBu, 0.63 g, 6.50 mmol) were dissolved in 50 mL of toluene. Subsequently, tris(dibenzylideneacetone)dipalladium(0) (Pd_2_(dba)_3_, 0.20 g, 0.22 mmol) and tri-tert-butylphosphine ((tBu)_3_P, 0.04 g, 0.22 mmol) were introduced under a nitrogen atmosphere. The resulting mixture was stirred at 110 °C for 2.5 h. Upon completion of the reaction, the mixture was cooled, poured into water, and extracted with dichloromethane. The organic layer was dried over MgSO_4_ and concentrated under reduced pressure. The resulting crude material was then purified by silica gel column chromatography (MC:n-hexane = 1:9, *v*/*v*), affording a white solid with a 77% yield. ^1^H NMR (400 MHz, DMSO-d6) δ 8.51 (s, 2H), 8.37–8.35 (d, *J* = 8.5 Hz, 1H), 8.31–8.32 (d, *J* = 7.7 Hz, 1H), 8.06–8.04 (d, *J* = 8.1 Hz, 1H), 7.81–7.78 (d, *J* = 8.6 Hz, 1H), 7.69–7.67 (d, *J* = 8.5 Hz, 2H), 7.47–7.16 (m, 10H), 1.42 (s, 18H); HR-MS(EI, *m*/*z*): [M+] calc’d for C_42_H_36_BNO_2_, 597.2833; found, 597.2800; elemental analysis calc’d (%) for C_70_H_52_N_2_O_2_: C 84.49, H 6.11, N 2.22, O 5.11; found: C 84.42, H 6.07, B 1.81, N 2.34, O 5.35.

#### 2.1.4. Synthesis of 9-(2,12-Di-tert-butyl-5,9-dioxa-13b-boranaphtho[3,2,1-de]anthracen-7-yl)-9H-carbazole (TDBA-Cz) (**4**)

Under N_2_ conditions, Compound **2** (1.00 g, 2.17 mmol), 9H-carbazole (0.44 g, 0.26 mmol), and sodium tert-butoxide (NaOtBu, 0.63 g, 6.50 mmol) were dissolved in 50 mL of toluene. Pd_2_(dba)_3_ (0.20 g, 0.22 mmol) and tri-tert-butylphosphine ((tBu)_3_P, 0.04 g, 0.22 mmol) were subsequently introduced. Stirring was continued at 110 °C for 2.5 h, after which the reaction mixture was allowed to cool to room temperature, poured into water, and extracted with dichloromethane. The organic extracts were dried over anhydrous MgSO_4_, concentrated under vacuum, and purified by silica gel column chromatography (MC:hexane = 1:9, *v*/*v*), yielding a white solid in 85% yield. ^1^H NMR (400 MHz, DMSO-d6) δ 8.60 (s, 2H), 8.26–8.24 (d, *J* = 8.5 Hz, 2H), 7.82–7.80 (d, *J* = 8.4 Hz, 2H), 7.63–7.61 (d, *J* = 8.4 Hz, 2H), 7.50–7.42 (m, 6H), 7.33–7.29 (m, 2H), 1.43 (s, 18H); HR-MS(EI, *m*/*z*): [M+] calc’d for C_38_H_34_BNO_2_, 547.2683; found, 547.2700; elemental analysis calc’d (%) for C_70_H_52_N_2_O_2_: C 83.48, H 6.24, N 2.31, O 5.42; found: C 83.36, H 6.26, B 1.97, N 2.56, O 5.84.

## 3. Results and Discussion

### 3.1. Molecular Design, Synthesis, and Characterization

**Scheme 1:**
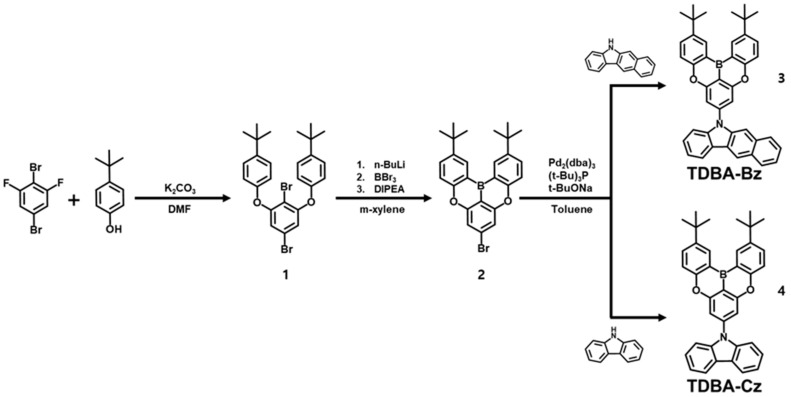
Synthetic of TDBA-Bz and TDBA-Cz.

**Figure 1:**
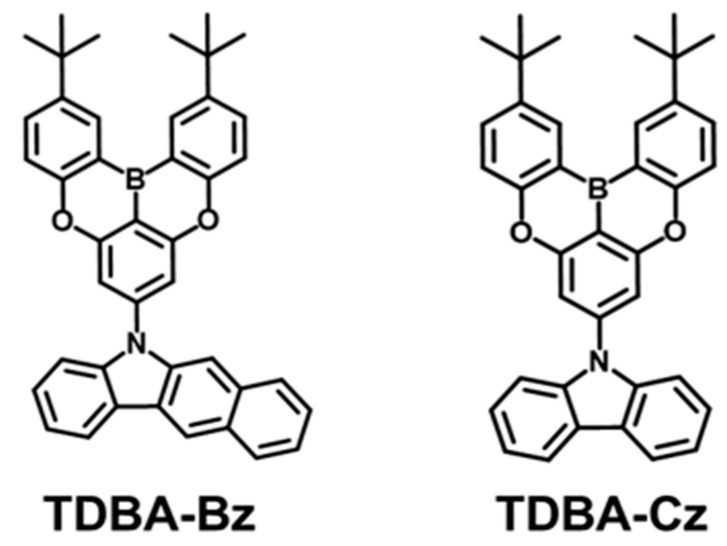
Chemical structures of TDBA-Bz and TDBA-Cz.

Due to its outstanding emission efficiency and narrow FWHM, DOBNA has attracted considerable attention as a blue-emitting organic material [30,31,32,33]. In particular, our research group previously reported an outstanding electroluminescent performance of 36.2% by employing TDBA, a core structure in which tert-butyl groups are substituted at the 2- and 10-positions of DOBNA [34]. The two tert-butyl groups substituted on the TDBA core are expected to reduce intermolecular interactions, thereby stabilizing the intrinsic optical properties during film formation and device fabrication. In this study, we synthesized and prepared two violet-blue organic emitters, TDBA-Bz and TDBA-Cz, by introducing Bz and Cz at the 7-position of the TDBA moiety (Scheme 1 and Figure 1). The two synthesized compounds are expected to exhibit high thermal stability and short-wavelength emission due to the rigid structures of both the TDBA core and the incorporated Bz and Cz units. In particular, TDBA-Cz possesses a shorter π-conjugation length compared to TDBA-Bz, which is anticipated to enable the fabrication of high-purity violet-blue emitting devices. Both Bz and Cz, used as donor units, exhibit excellent electron-donating properties and were strategically incorporated into the TDBA core to form donor–acceptor-type structures. This structural approach maintains the lowest unoccupied molecular orbital (LUMO) predominantly on the TDBA while adjusting the highest occupied molecular orbital (HOMO) energy level through the electron-donating properties of the donor moieties. As a result, charge transfer (CT) characteristics can be effectively induced. TDBA-Bz and TDBA-Cz were synthesized through a series of reactions, including nucleophilic substitution, intramolecular electrophilic borylation, and Buchwald–Hartwig coupling. The synthesized compounds were purified through column chromatography and their structures were confirmed by nuclear magnetic resonance (NMR) spectroscopy, mass spectrometry, and elemental analysis.

### 3.2. Photophysical Properties

**Figure 2:**
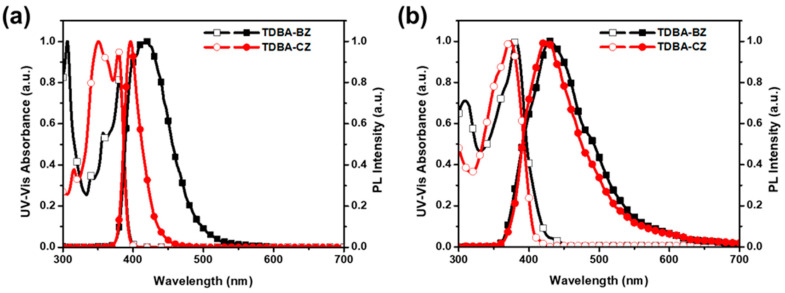
UV-vis absorption and PL spectra of TDBA-Bz and TDBA-Cz: (**a**) solution state, toluene, 1 × 10^−5^ M, UV-vis absorption, and PL spectra; (**b**) vacuum-deposited film (thickness: 50 nm), UV-vis absorption, and PL spectra.

To investigate the photophysical properties, TDBA-Bz and TDBA-Cz were measured using UV–visible (UV–vis) absorption and photoluminescence (PL) spectra in both solution and film states, as presented in Figure 2, and Table 1 summarizes the optical properties and other photophysical parameters. In solution, the maximum UV–vis absorption peaks (UV_max_) of TDBA-Bz and TDBA-Cz occurred at 381 nm and 379 nm, respectively, attributed to short-range CT transitions. In the absorption spectra, peaks below 320 nm are attributed to π–π* transitions, whereas the peaks observed around 350 nm are ascribed to n–π* transitions derived from the Bz and Cz fragments [35]. In solution, the PL maximum wavelengths (PL_max_) of TDBA-Bz and TDBA-Cz were observed at 420 nm and 396 nm. FWHM values were 68 nm for TDBA-Bz and 28 nm for TDBA-Cz. The remarkably narrow FWHM of TDBA-Cz indicates that molecular rotation and vibration are effectively suppressed in solution due to its rigid molecular structure. PL_max_ of TDBA-Bz and TDBA-Cz in the film state was observed at 429 nm and 422 nm, respectively, indicating a slightly bathochromic shift compared to their solution-state counterparts. The PL data in both solution and film states exhibited maximum emission within the range of 400–430 nm. This emission range corresponds to the violet-blue region and falls within the applicable wavelength range for blue-emitting materials. In thin films, TDBA-Bz and TDBA-Cz showed FWHM values of 100 nm and 84 nm, respectively. The increased planarity of the molecular structures strengthened intermolecular interactions, thereby inducing broader emissions and red-shifted PL characteristics. As shown in Figure S1, individual analysis of the two materials’ graphs revealed that, although TDBA-Bz and TDBA-Cz exhibited comparable PL shapes and maximum wavelengths in the film state, a clear 16 nm gap in the FWHM was detected. This confirms that vibronic variations due to the expanded π-conjugation arise in TDBA-Bz. The increase in FWHM observed in the film state is attributed to molecular rotation and vibration associated with the presence of single bonds within the molecule, a phenomenon commonly reported in other studies on thermally activated delayed fluorescence (TADF) materials. Therefore, the broadening of the FWHM is considered to result from vibronic peaks rather than excimer formation [36,37,38]. Additionally, it was confirmed that the PL spectral shapes and maximum wavelengths of TDBA-Bz and TDBA-Cz differ in the solution state. Since the synthesized materials are applied as dopants in the OLED devices developed in this study, and the optical properties of the dopants are closely related to their optical characteristics in solution, the distinct optical and electrical properties of the two materials have been identified.

TDBA-Bz and TDBA-Cz exhibited singlet excited state energies (S_1_) of 3.25 eV and 3.28 eV, and triplet state energies (T_1_) were determined to be 3.03 eV for TDBA-Bz and 3.20 eV for TDBA-Cz, with TDBA-Cz demonstrating a ~0.17 eV higher T_1_ energy level (Table 2). Consequently, the ΔE_ST_ values were calculated as 0.22 eV for TDBA-Bz and 0.08 eV for TDBA-Cz. PL spectra measured at 298 K and 77 K were used to estimate the S_1_ and T_1_ energy levels of both compounds. The observed small ΔE_ST_ values for both compounds are mainly due to the twist geometry between the donor and acceptor moiety. TDBA-Bz and TDBA-Cz showed photoluminescence quantum yields (PLQYs) of 31%/12% and 69%/11% in solution and evaporated film states, with TDBA-Cz demonstrating overall higher PLQY values. The high PLQY of TDBA-Cz in solution is particularly advantageous for achieving high electroluminescent efficiency in solution-processed devices. Additionally, to further investigate the emission processes, time-resolved photoluminescence (TRPL) analyses were performed on 30 wt% TDBA-Bz- and TDBA-Cz-doped 1,3-bis(N-carbazolyl)benzene (mCP) films prepared by spin-coating (Figure S2). The delayed fluorescence lifetimes (τ_d_) were determined to be 10.5 μs and 22.7 μs, while the prompt fluorescence lifetimes (τ_p_) were measured to be 3.96 ns for TDBA-Bz and 6.04 ns for TDBA-Cz. The calculated radiative decay rate constants (*k_rad_*) were 3.78 × 10^7^ s^−1^ for TDBA-Bz and 5.45 × 10^7^ s^−1^ for TDBA-Cz, indicating a relatively higher *k_rad_* for TDBA-Cz (Table S1). The non-radiative rate constants (*k_nr_*) were 5.37 × 10^7^ s^−1^ and 2.77 × 10^7^ s^−1^ for TDBA-Bz and TDBA-Cz. Compared to TDBA-Bz, TDBA-Cz exhibited a higher *k_rad_* and a lower *k_nr_*, resulting in an improved *k_rad_*/*k_nr_* ratio of 1.98, compared to 0.704 for TDBA-Bz. The reverse intersystem crossing rate constants (*k_RISC_*) were calculated to be 2.23 × 10^3^ s^−1^ for TDBA-Bz and 4.79 × 10^3^ s^−1^ for TDBA-Cz. Although TDBA-Cz, with its relatively smaller ΔE_ST_, exhibited a slightly faster RISC rate, both compounds showed slower RISC rates compared to typical TADF materials [34,39,40]. Based on molecular orbital calculations, the spin–orbit coupling (SOC) strengths of S_1_-T_1_ states were found to be relatively small, calculated as 0.18 for TDBA-Bz and 0.12 for TDBA-Cz. These results suggest that the contribution of the RISC process to the overall emission is limited, and that prompt emission serves as the main pathway determining the emission efficiency. Ultraviolet photoelectron spectroscopy (AC-2) was employed to evaluate the HOMO energy levels of TDBA-Bz and TDBA-Cz, and the LUMO energy levels were subsequently estimated based on the obtained HOMO values and the optical band gaps. The optical band gaps were derived from the absorption onset, determined through (*hν*) versus (*αhν*)^2^ plots, with α representing the absorption coefficient, h being Planck’s constant, and ν denoting the frequency of the incident photons. TDBA-Bz showed HOMO and LUMO values of −5.69 eV and −2.78 eV, respectively, and TDBA-Cz exhibited corresponding values of −5.79 eV and −2.75 eV, resulting in optical band gaps of 2.91 eV and 3.04 eV.

### 3.3. Theoretical Calculation

**Figure 3:**
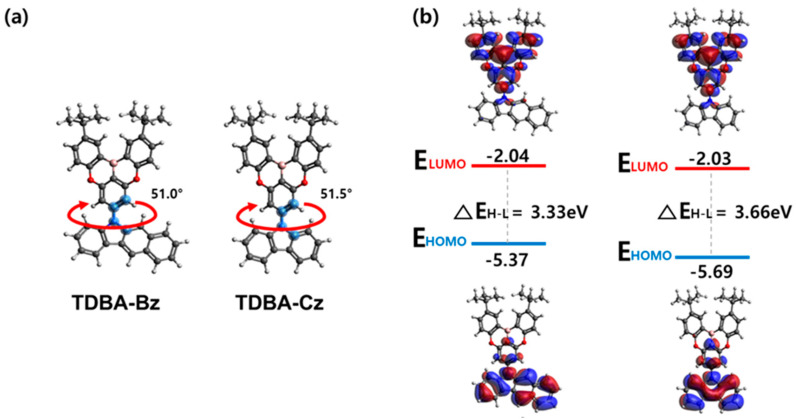
(**a**) The optimized molecular geometries and dihedral angles of TDBA-Bz and TDBA-Cz. (**b**) A visualization of the frontier molecular orbitals along with the HOMO and LUMO energy levels for TDBA-Bz and TDBA-Cz, calculated at the B3LYP/def2-TZVPP level.

The molecular structures of TDBA-Bz and TDBA-Cz were optimized using density functional theory (DFT) at the B3LYP/def2-TZVPP level, and their dihedral angles were evaluated. The distributions of HOMO and LUMO electron densities are depicted in Figure 3. As shown in Figure 3a, the dihedral angles between the donor and acceptor units were found to be 51.0° for TDBA-Bz and 51.5° for TDBA-Cz, indicating comparable torsional characteristics in both molecules. Such twisted angles are expected to effectively suppress intermolecular stacking, thereby enhancing solubility and making these compounds promising candidates for solution-processable devices. As shown in Figure 3b, the LUMO electron density is primarily localized on the TDBA core, whereas the HOMO orbitals of TDBA-Bz and TDBA-Cz are largely distributed over the Bz and Cz side groups, respectively. This distribution reflects the strong electron-donating characteristics of the peripheral units, imparting a pronounced CT character to both molecules. The calculated LUMO energy levels for TDBA-Bz and TDBA-Cz were found to be −2.04 eV and −2.03 eV, respectively, while their HOMO levels were determined as −5.37 eV and −5.69 eV. The resulting band gaps (ΔE_H−L_) were 3.33 eV for TDBA-Bz and 3.66 eV for TDBA-Cz, exhibiting a trend consistent with the experimentally derived energy values. The increase in the HOMO level of TDBA-Bz is explained by the extended delocalization and electron-rich characteristics of the Bz unit, causing the energy level to shift closer to the vacuum level. The S_1_, T_1_, and ΔE_ST_ values of TDBA-Bz and TDBA-Cz calculated via DFT were 2.93/2.52/0.41 eV and 3.22/2.89/0.33 eV, respectively, showing trends consistent with the experimentally measured results (Table S2). Additionally, the oscillator strength values of TDBA-Bz and TDBA-Cz were also calculated (Table S3). Around the UV_max_ absorption region at 380 nm, TDBA-Cz exhibited an oscillator strength of 0.25, approximately 1.47 times greater than that of TDBA-Bz (0.17), reflecting a stronger light absorption capability. The enhanced emission efficiency of TDBA-Cz is further supported by the PLQY results obtained from photophysical characterization, which exhibit a consistent trend.

### 3.4. Thermal Properties

**Figure 4:**
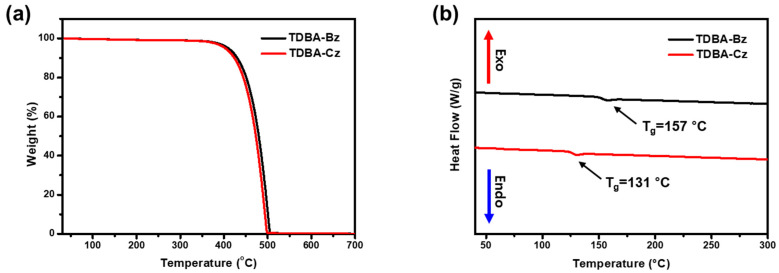
(**a**) TGA and (**b**) DSC curves for TDBA-Bz and TDBA-Cz.

As shown in Figure 4, the degradation temperatures (T_d_) and glass transition temperatures (T_g_) of TDBA-Bz and TDBA-Cz were evaluated by Thermal Gravimetric Analysis (TGA) and Differential Scanning Calorimetry (DSC). According to the TGA, the T_d_ values, corresponding to 5% weight loss, were measured as 505 °C for TDBA-Bz and 496 °C for TDBA-Cz. The T_g_ was measured to be 157 °C for TDBA-Bz and 131 °C for TDBA-Cz. No clear melting points were observed for either compound up to 300 °C. The high T_g_ above 130 °C further confirms the excellent thermal stability of these materials [41,42,43].

### 3.5. Electroluminescence Properties

**Figure 5:**
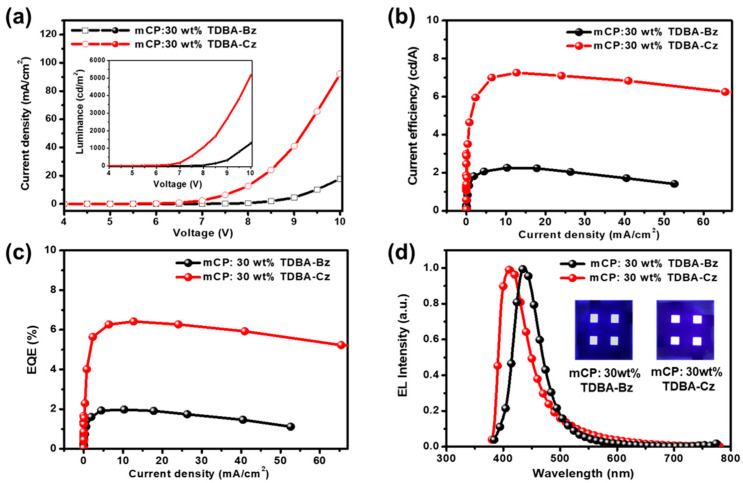
(**a**) Current density–voltage (J–V) curves of the solution-processed doped OLED devices for TDBA-Bz and TDBA-Cz in mCP (inset: luminance–voltage curves), (**b**) current efficiency–current density characteristics, (**c**) EQE–current density characteristics, and (**d**) EL spectra of the corresponding devices (inset: photographs of devices operating at 10 V.).

OLED devices were fabricated using a hybrid solution–evaporation process to investigate the suitability of TDBA-Bz and TDBA-Cz as blue dopant materials. Prior to device fabrication, in order to evaluate the state of the films, the two materials were doped at 30 wt% into an mCP host and spin-coated, followed by optical microscopy observation. Figure S3 demonstrates that TDBA-Cz produced relatively fewer dark spots than TDBA-Bz, indicating the formation of a smoother and cleaner film. Both films exhibited violet-blue emission (Figure S4). TDBA-Bz is a newly developed material, synthesized and characterized for the first time in this study, whereas TDBA-Cz was previously reported by the Hu. J et al. group in 2024. However, in an earlier study, TDBA-Cz-based devices were fabricated via vacuum deposition, while in this study, devices were fabricated using a solution process, achieving significantly superior performance compared to the previously reported results [44]. The EL characteristics of the devices are presented in Figure 5 and Table 3. The device structure was composed of ITO (anode, 150 nm)/poly(3,4-ethylenedioxythiophene) polystyrene sulfonate (PEDOT:PSS) (solution-processed, 40 nm)/poly(9-vinylcarbazole) (PVK) (solution-processed, 20 nm)/mCP: 30 wt% TDBA-B_Z_ or TDBA-C_Z_ (solution-processed, 20 nm)/1,3,5-tris(1-phenyl-1H-benzimidazol-2-yl)benzene (TPBi) (evaporated, 40 nm)/LiF (evaporated, 1 nm)/Al (cathode, 200 nm). PEDOT:PSS and PVK were used as the hole injection layer (HIL) and hole transport layer (HTL). In the EML, mCP served as the host material, with TDBA-Bz and TDBA-Cz acting as the dopants. The deposited TPBi layer simultaneously acted as the hole-blocking and electron-transporting layers in doped device structure (Figure S5). The host material mCP, which has a wide band gap, was used in combination with the dopants TDBA-Bz and TDBA-Cz. Analysis of the dopants’ absorbance spectra in solution and the host’s PL spectrum in the film state revealed sufficient spectral overlap, verifying efficient energy transfer from the host to the dopant (Figure S6). OLED devices fabricated using TDBA-Bz and TDBA-Cz as dopants exhibited similar turn-on voltages of 6.79 V and 6.82 V. The driving voltages at a current density of 10 mA/cm^2^ were 9.48 V for TDBA-Bz and 7.75 V for TDBA-Cz. This might be attributed to the lower carrier transport ability of TDBA-Bz. The maximum current efficiency (CE_max_) values were 2.26 cd/A and 7.25 cd/A, and the EQE_max_ values were 1.98% and 6.45%. The doped device using TDBA-Cz as the dopant exhibited higher luminous efficiency. This can be attributed to the high oscillator strength and PLQY values of the TDBA-Cz dopant, as observed in the photophysical properties. TDBA-Bz exhibited a roll-off of 14.6% at a brightness of 2000 cd/m^2^, relative to its EQE_max_. In contrast, TDBA-Cz showed a smaller roll-off of 4.81% at the same brightness of 2000 cd/m^2^ (Figure S7). This performance can be attributed to the bipolar characteristics of the synthesized molecular structure, which leads to optimized injection of both electrons and holes, resulting in stable device operation. Comparison with our previous work, in which TAT and T-TAT were used as dopants in solution-processed EL devices, revealed significantly improved performance. The EL device using TDBA-Cz as the dopant in a solution-processed system exhibited a blue shift of about 40 nm in the EL spectrum and an increase in current efficiency of more than eight times compared to the previously reported materials [45]. The CIE (x, y) values of the two devices were (0.181, 0.114) for TDBA-Bz and (0.167, 0.086) for TDBA-Cz, with EL_max_ values of 436 nm and 413 nm, respectively, confirming violet-blue emission. This study presents two dopant materials, TDBA-Bz and TDBA-Cz, that can be used for device fabrication through a hybrid solution–evaporation process. Both materials demonstrate low roll-off and high EQE values, making them high-purity violet-blue emitters with promising potential for large-area OLED production in the future.

## 4. Conclusions

In this study, TDBA-Bz and TDBA-Cz, TADF dopants combining the high rigidity of DOBNA-based TDBA with carbazole derivatives, were designed and synthesized. TDBA-Bz and TDBA-Cz demonstrated high color-purity violet-blue emission characteristics in both solution and film states and showed excellent thermal stability. TDBA-Cz exhibited a higher PLQY of 69% in solution compared to TDBA-Bz. In EL devices fabricated through the hybrid solution–evaporation process, TDBA-Bz and TDBA-Cz had EQE values of 1.98% and 6.45%, with EL wavelengths of 436 nm and 413 nm. In particular, TDBA-Cz achieved approximately three times the EQE_max_ of TDBA-Bz and exhibited violet-blue emission with a CIE value of 0.086. Additionally, at a brightness of 2000 cd/m^2^, TDBA-Cz demonstrated a low roll-off of only 4.81%, showing excellent stability even at high brightness. The enhancement is ascribed to the balanced transport of holes and electrons achieved through the bipolar molecular structure, which efficiently controls the exciton recombination region. The two dopants proposed in this study possess molecular structures suitable for solution processing, along with excellent emission properties and device driving stability. Therefore, they are expected to be promising candidate materials for the realization of large-area OLED displays in the future.

## Figures and Tables

**Table 1:** Photophysical properties of synthesized compounds.

Compound	Solution ^a^		Film ^b^		PLQY ^a,b^ (%)	*k_rad_* ^c^ (10^7^ s^−1^)	*k_nr_* ^c^ (10^7^ s^−1^)	*k_rad_*/*k_nr_*
	UV_max_ (nm)	PL_max_ (FWHM) (nm)	UV_max_ (nm)	PL_max_ (FWHM) (nm)				
TDBA-BZ	306, 340, 357, 381	420 (67)	309, 381	429 (100)	31/12	3.78	5.37	0.70
TDBA-CZ	315, 350, 379	396 (28)	373	422 (84)	69/11	5.45	2.77	1.98

^a^ 10^−5^ M toluene solution, ^b^ film thickness: 50 nm, and ^c^ solution processed 30 wt% doped film.

**Table 2:** Photophysical and electronic characteristics of synthesized compounds.

Compound	E_S_/E_T_^a^ (eV)	ΔE_ST_^b^ (eV)	HOMO ^c^ (eV)	LUMO (eV)	Band Gap (eV)
TDBA-BZ	3.25/3.03	0.22	−5.69	−2.78	2.91
TDBA-CZ	3.28/3.20	0.08	−5.79	−2.75	3.04

^a^ singlet and triplet energy levels were measured in toluene, ^b^ ΔE_ST_ was calculated according to the difference between E_S_ and E_T_, and ^c^ the HOMO level was measured by AC-2.

**Table 3:** EL performances of the OLED doped devices at 10 mA/cm^2^.

EML	V_on_^a^ (V)	CE ^b^ (cd/A)		EQE ^c^ (%)		CIE (x, y) ^d^	EL_max_ (nm)	FWHM (nm)
		Max	2000 nit	Max	2000 nit			
mCP: 30 wt% TDBA-BZ	6.79	2.26	1.96	1.98	1.69	(0.181, 0.114)	436	55
mCP: 30 wt% TPDBA-CZ	6.82	7.25	6.98	6.45	6.14	(0.167, 0.086)	413	59

^a^ Turn-on voltage at 1 cd/m^2^, ^b^ current efficiency, ^c^ external quantum efficiency, and ^d^ CIE color coordinates at 7 V.

## Data Availability

The data are contained within the article and Supplementary Materials.

## Supplementary Materials

The following supporting information can be downloaded at https://www.mdpi.com/article/10.3390/ma18102213/s1: Figure S1. UV–vis absorption and photoluminescence (PL) spectra of TDBA-Bz and TDBA-Cz. (a, b) Measurements in toluene (1.0 × 10^−5^ M). (c, d) Spectra of vacuum-deposited thin films (50 nm) (inset: the FWHM values of the PL spectra). Figure S2. Transient photoluminescence decay spectra of doped films (spin-coated mCP doped with 30% emitters). Table S1. Time-resolved photoluminescence (TRPL) parameters of mCP-doped (30 wt%) TDBA-Bz and TDBA-Cz films. Table S2. Calculated singlet/triplet excited states and spin–orbit coupling (SOC) values of TDBA-Cz and TDBA-Bz. Table S3. Oscillator strength of TDBA-Cz and TDBA-Bz (calculated at the B3LYP/def2-TZVPP). Figure S3. Optical microscope images (20× magnification) of spin-coated films measured using an OLYMPUS STM6: (a) TDBA-Bz and (b) TDBA-Cz. Figure S4. UV-excited emission images of spin-coated doped films (30 wt% of emitters in mCP host): (a) TDBA-Bz and (b) TDBA-Cz. Figure S5. (a) Molecule structures and (b) energy level diagrams used in solution-processed OLEDs. Figure S6. Photoluminescence (PL) spectrum of spin-coated mCP film and UV–vis absorption spectra of TDBA-Bz and TDBA-Cz measured in toluene solution. Figure S7. External quantum efficiency (EQE) versus luminance for doped films (spin-coated mCP films doped with 30 wt% emitters). Figure S8. ^1^H NMR of 4,4′-((2,5-dibromo-1,3-phenylene)bis(oxy))bis(tert-butylbenzene). Figure S9. ^1^H NMR of 7-bromo-2,12-di-tert-butyl-5,9-dioxa-13b-boranaphtho[3,2,1-de]anthracene. Figure S10. ^1^H NMR of 5-(2,12-di-tert-butyl-5,9-dioxa-13b-boranaphtho[3,2,1-de]anthracen-7-yl)-5H-benzo[b]carbazole (TDBA-Bz). Figure S11. ^1^H NMR of 9-(2,12-di-tert-butyl-5,9-dioxa-13b-boranaphtho[3,2,1-de]anthracen-7-yl)-9H-carbazole (TDBA-Cz). Figure S12. HR-Mass spectroscopy: (a) TDBA-Bz and (b) TDBA-Cz.
