# Supplementary material for: High-Efficiency Deep-Blue Solution-Processed OLED Devices Enabled by New Dopant Materials
Source: Materials (Basel). 2025 May 10;18(10):2213. doi: 10.3390/ma18102213 (PMC12113092; doi:10.3390/ma18102213)
Supplement: Supplementary file 1 [file materials-18-02213-s001.zip › materials-3609701-supplementary.pdf]

Supplementary Information

High-Efficiency Deep Blue Solution-Processed OLED Devices Enabled by New Dopant Materials

Saeyoung Oh [1, †], Hyukmin Kwon [1, †], Sangwook Park[1], Seokwoo Kang[1], Sang-Tae Kim[1], Kiho Lee[1], Hayoon Lee[1], Jongwook Park[1,*]

Integrated Engineering, Department of Chemical Engineering, Kyung Hee University, Yongin, Gyeonggi, 17104, Republic of Korea; tpdud5821@khu.ac.kr(S.O.); hm531@khu.ac.kr(H.K.); pswook@khu.ac.kr(S.P.); swkang@khu.ac.kr(S.K.); kimst2@khu.ac.kr(S.K.); kiholee@khu.ac.kr(K.L.); kssarang1@khu.ac.kr(H.L.)

Corresponding authors. E-mail addresses: Jongpark@khu.ac.kr (J. Park)

[†]Saeyoung Oh and Hyukmin Kwon contributed equally to this work as the first coauthor.

1. General Information

The reagents and solvents used in this study were purchased from Sigma-Aldrich, Tokyo Chemical Industry (TCI), or Alfa Aesar, with a minimum purity of 98 %, and were used as received without further purification. Analytical TLC was carried out on a Merck 60 F254 silica gel plate, and column chromatography was performed on Merck 60 silica gel (230-400 mesh, Burlington, MA, USA). ^{1}H NMR spectra were obtained in chloroform-d and dimethyl sulfoxide-d6 (DMSO-d6) using a JNM-ECZ400S/L1 spectrometer (JEOL, Tokyo, Japan) at room temperature. High-resolution mass spectrometry (HRMS) was conducted via electron ionization (EI) on a JMS-700, 6890 Series mass spectrometer (JEOL, Tokyo, Japan). Ultraviolet–visible (UV–vis) absorption spectra were recorded using a UV-1900i UV/Vis/NIR spectrophotometer (Shimadzu, Kyoto, Japan). Photoluminescence (PL) spectra were measured using a PerkinElmer LS55 spectroflu-orometer equipped with a xenon flash lamp (PerkinElmer, Inc., Waltham, MA, USA). Absolute photoluminescence quantum yields (PLQYs) were determined using a Ha-mamatsu Quantaurus-QY C11347 system (Hamamatsu Photonics, Shizuoka-ken, Ja-pan). The glass transition temperatures (T_g) and melting temperatures (T_m) of the compounds were obtained on a Differential Scanning Calorimetry (DSC) Discovery DSC 26 instrument (TA Instruments, New Castle, DE, USA) under a nitrogen atmosphere. compounds were heated to 300 °C at a rate of 5 °C/min, then cooled at the same rate, and subsequently reheated under the same conditions as used in the initial heating process. Degradation temperatures (T_d) values of the compounds were measured with Thermal Gravimetric Analysis (TGA) using a SDT Q600 system (TA Instruments, New Castle, DE, USA). Samples were heated to 800 ℃ at a rate of 10 ℃/min. The highest occupied molecular orbital (HOMO) energy levels were determined with ultraviolet photoelectron spectroscopy (Riken Keiki AC-2, Nara, Japan). The lowest unoccupied molecular orbital (LUMO) energy levels were derived from the HOMO energy levels and the band gaps. For the EL devices, all organic layers were deposited under 10^{-6} torr, with a rate of deposition of 1 Å/s to give a deposition area of 4 mm^2. Time-resolved photoluminescence (TRPL) decay time values were obtained by using Quantaurus Tau (Hamamatsu Photonics, Shizuoka-ken, Japan). The thicknesses of the films were verified using ellipsometry. ITO glasses (25 mm × 25 mm) were ultrasonically washed in, sequentially, acetone, ethanol, deionized water, and isopropyl alcohol and then dried in an oven at 80 °C. The ITO substrates were further cleaned with a UV–ozone cleaner for 10 min before the spin-coating process. In doped devices, a 40 nm-thick hole injection layer (HIL) was prepared by spin-coating using poly(3,4-ethylenedioxythiophene) polystyrene sulfonate (PEDOT:PSS, Clevios PVP AI4083) at 4000 rpm for 60 s and baking at 140 °C for 20 min. A 20 nm-thick hole transport layer (HTL) was prepared by spin-coating using poly(9-vinylcarbazole) (PVK, 6 mg/1 mL of chlorobenzene) at 3000 rpm for 40 s and baking at 150 °C for 30 min. After cooling to room temperature, the emitting materials (30 wt% in toluene) were spin-coated at 2000 rpm for 60 s. After annealing at 70 °C for 5 min, the prepared substrates were transferred to a thermal evaporation chamber. The chamber was evacuated to 2.0×10^{-5} torr, and a 40 nm-thick layer of 1,3,5-tris(1-phenyl-1H-benzimidazol-2-yl)benzene (TPBi, Lumtec, Taiwan), a 1 nm-thick layer of LiF, and a 200 nm-thick layer of Al were sequentially evaporated. The current density–voltage–luminance (J–V–L) characteristics of the fabricated EL devices were measured using a Keithley 2400 source meter (Tektronix, Cleveland, OH, USA), and luminance intensities were recorded using a Minolta CS-1000A spectrora-diometer (Konica Minolta, Tokyo, Japan)

2. *Supplementary figures and tables*

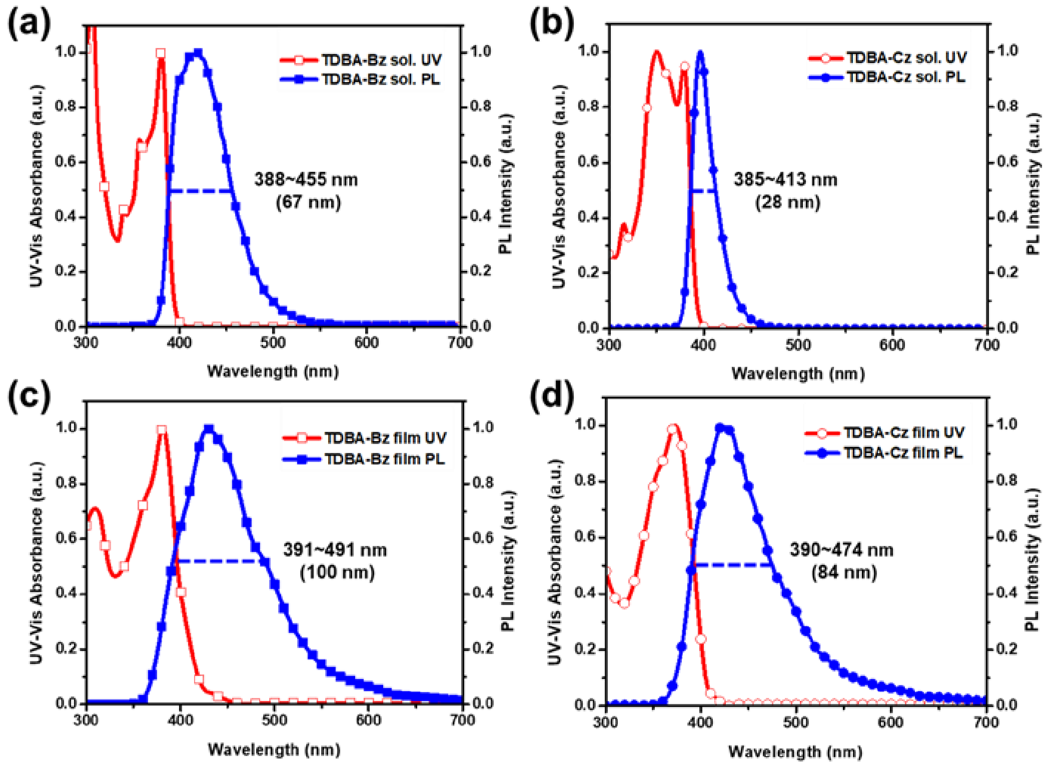

Fig. S1. UV–vis absorption and photoluminescence (PL) spectra of TDBA-Bz and TDBA-Cz. (a, b) Measurements in toluene (1.0 × 10^{-5} M). (c, d) Spectra of vacuum-deposited thin films (50 nm). (Inset: the FWHM values of the PL spectra)

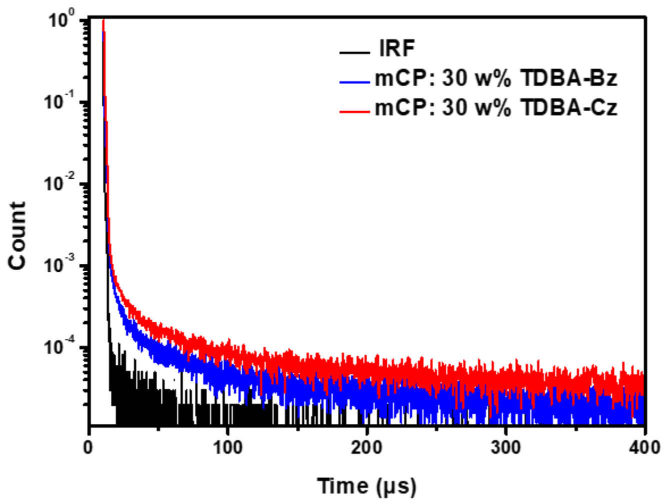

Fig. S2. Transient photoluminescence decay spectra of doped films (spin-coated mCP doped with 30 % emitters).

Table S1. Time-resolved photoluminescence (TRPL) parameters of mCP-doped (30 wt%) TDBA-Bz and TDBA-Cz films.

**Compound**	Φ_F	τ_p (ns)	τ_d (μs)	k_{rad} [c] (10^7 s^{-1})	k_{nr} [c] (10^7 s^{-1})	k_{rad}/k_{nr}	k_{RISC} (10^3 s^{-1})
**mCP: 30 wt% TDBA-BZ**	0.15	3.96	10.5	3.78	5.37	0.70	2.23
**mCP: 30 wt% TDBA-CZ**	0.33	6.04	22.7	5.45	2.77	1.98	4.79

Table S2. Calculated singlet/triplet excited states and spin–orbit coupling (SOC) values of **TDBA-Cz and TDBA-Bz.**

**Compound**	**T_1 (eV)**	**S_1 (eV)**	**ΔE_{ST} (eV)**	**SOC (cm^{-1})**
**TDBA-Bz**	2.52	2.93	0.41	0.18
**TDBA-Cz**	2.89	3.22	0.33	0.12

Table S3. Oscillator strength of **TDBA-Cz** and **TDBA-Bz** (calculated at the B3LYP/def2-TZVPP).

Compound	Absorption wavelength (nm)	Absorption energy (eV)	Oscillator strength	Characteristic of transition	Contribution (%)
**TDBA-Bz**	423.0	2.522	0.174749246	H → L+1	83.9
	364.7	2.813	0.047659214	H → L	87.6
	359.1	2.900	0.156628693	H-1 → L	95.4
	341.8	3.242	0.066299056	H-2 → L+1	86.0
**TDBA-Cz**	384.5	3.225	0.250890891	H → L	98.5
	359.1	3.453	0.160385275	H-1 → L	94.3
	342.8	3.617	0.000141416	H-2 → L	99.0

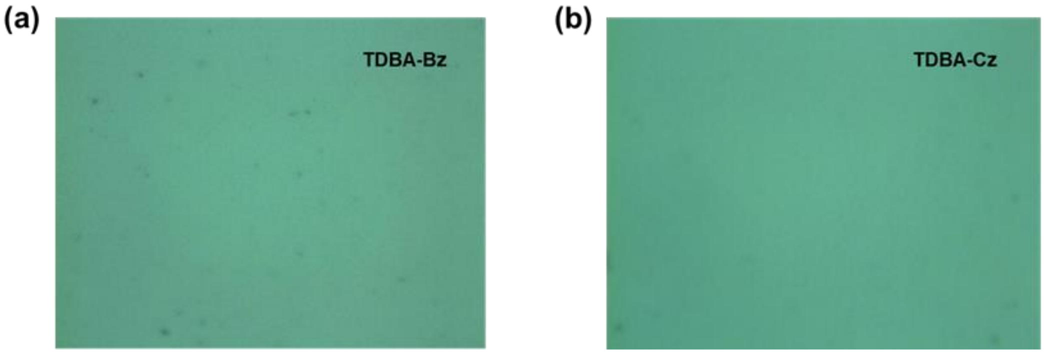

Fig. S3. Optical microscope images (20× magnification) of spin-coated films measured using an OLYMPUS STM6: (a) TDBA-Bz and (b) TDBA-Cz.

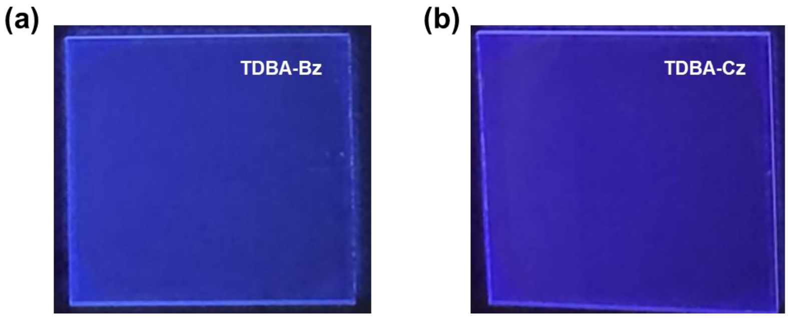

Fig. S4. UV-excited emission images of spin-coated doped films (30 wt% of emitters in mCP host): (a) TDBA-Bz and (b) TDBA-Cz.

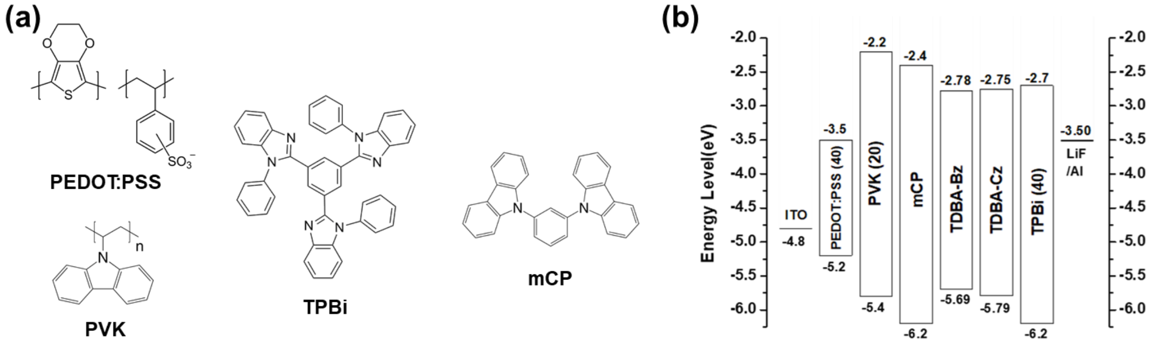

Fig. S5. (a) Molecule structures and (b) Energy level diagrams used in solution process OLEDs.

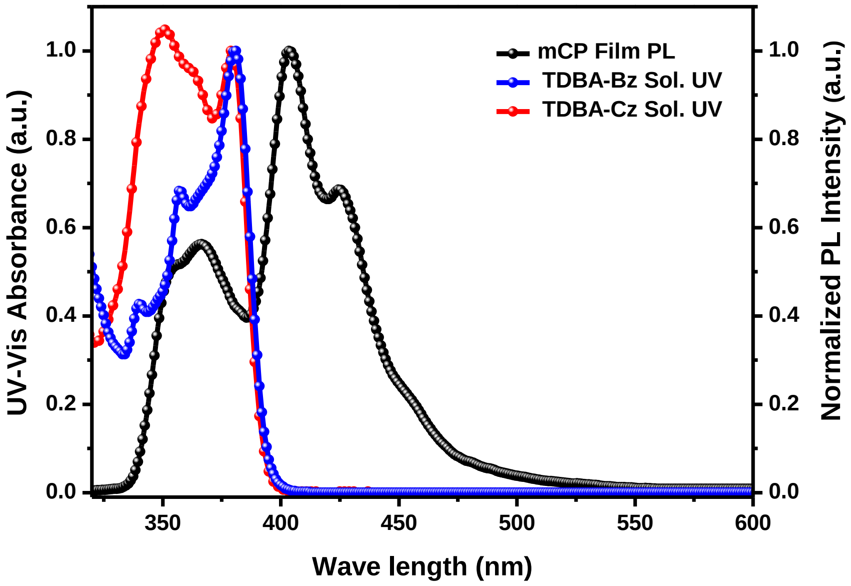

Fig. S6. Photoluminescence (PL) spectrum of spin-coated mCP film and UV–vis absorption spectra of TDBA-Bz and TDBA-Cz measured in toluene solution.

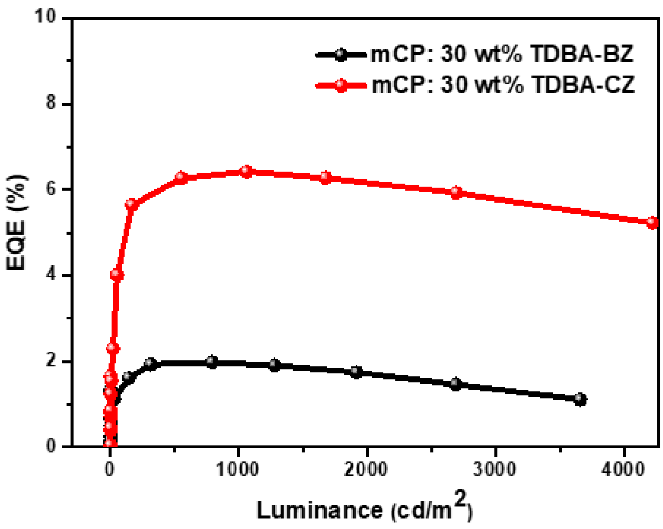

Fig. S7. External quantum efficiency (EQE) versus luminance for doped films (spin-coated mCP films doped with 30 wt% emitters).

2. NMR and HR-Mass

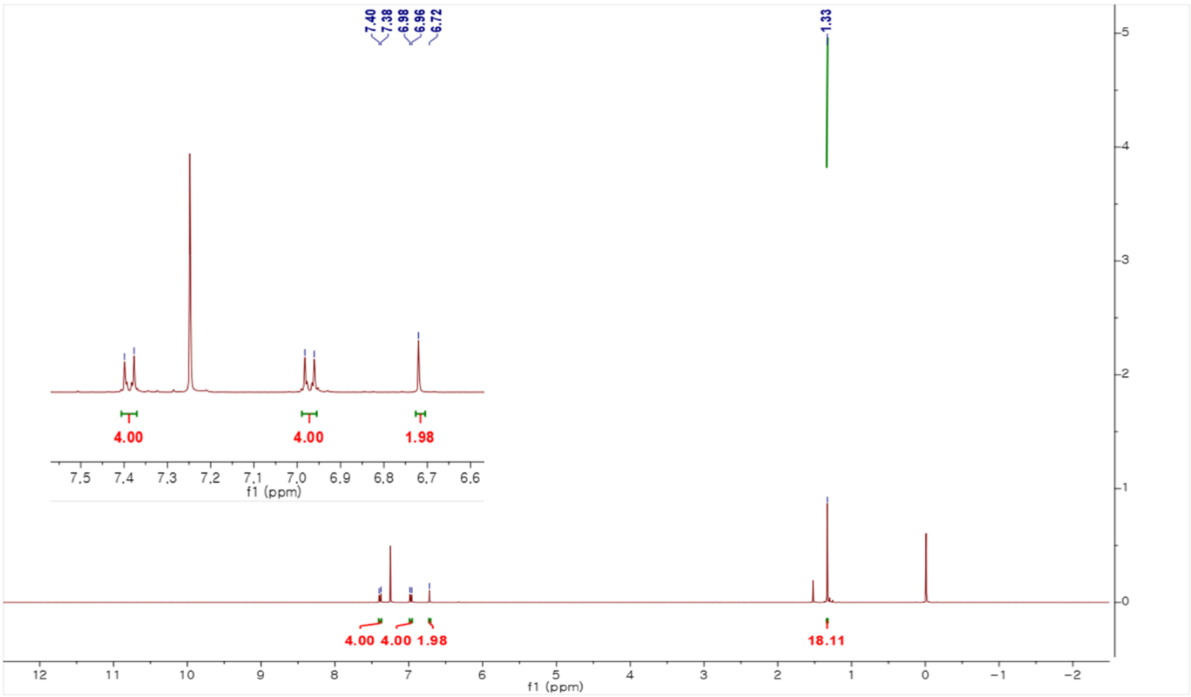

Fig. S8. ^{1}H NMR of **4,4'-((2,5-dibromo-1,3-phenylene)bis(oxy))bis(tert-butylbenzene)**

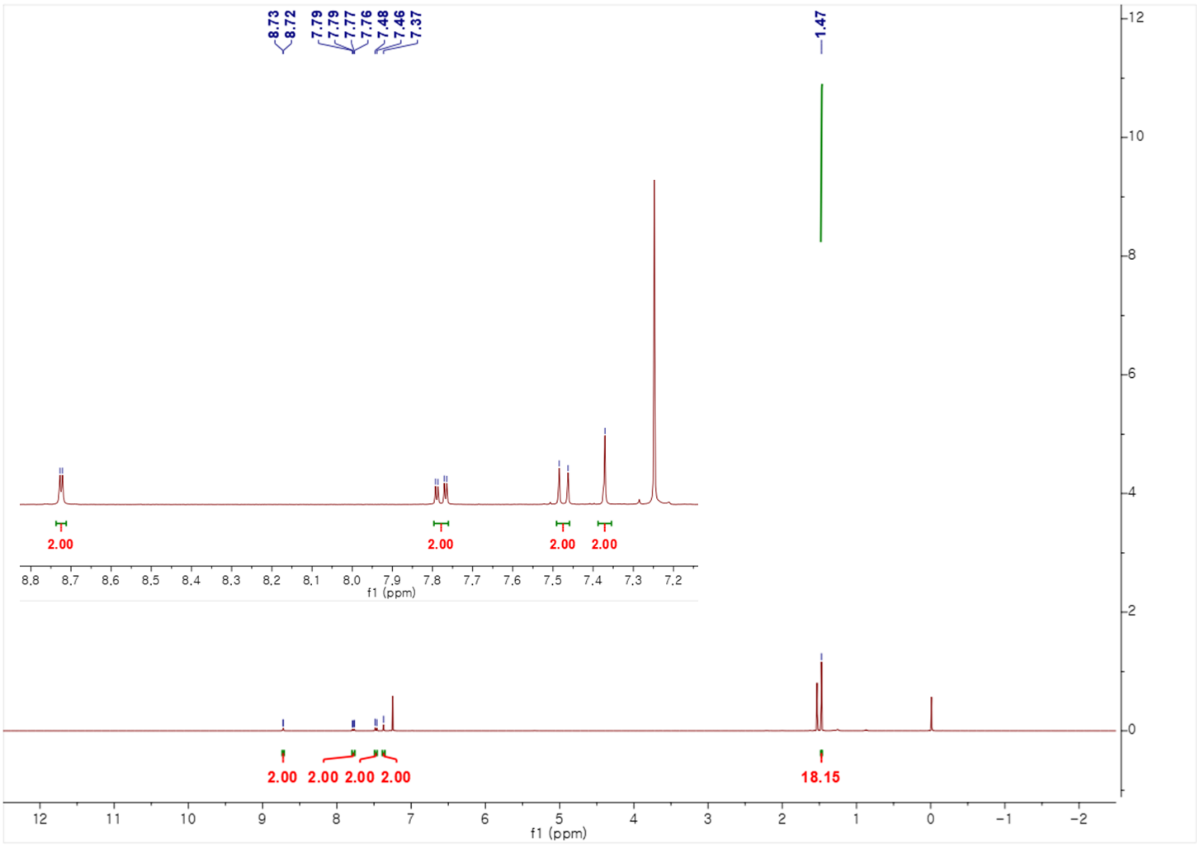

Fig. S9. ¹H NMR of **7-bromo-2,12-di-tert-butyl-5,9-dioxa-13b-boranaphtho[3,2,1-de]anthracene**

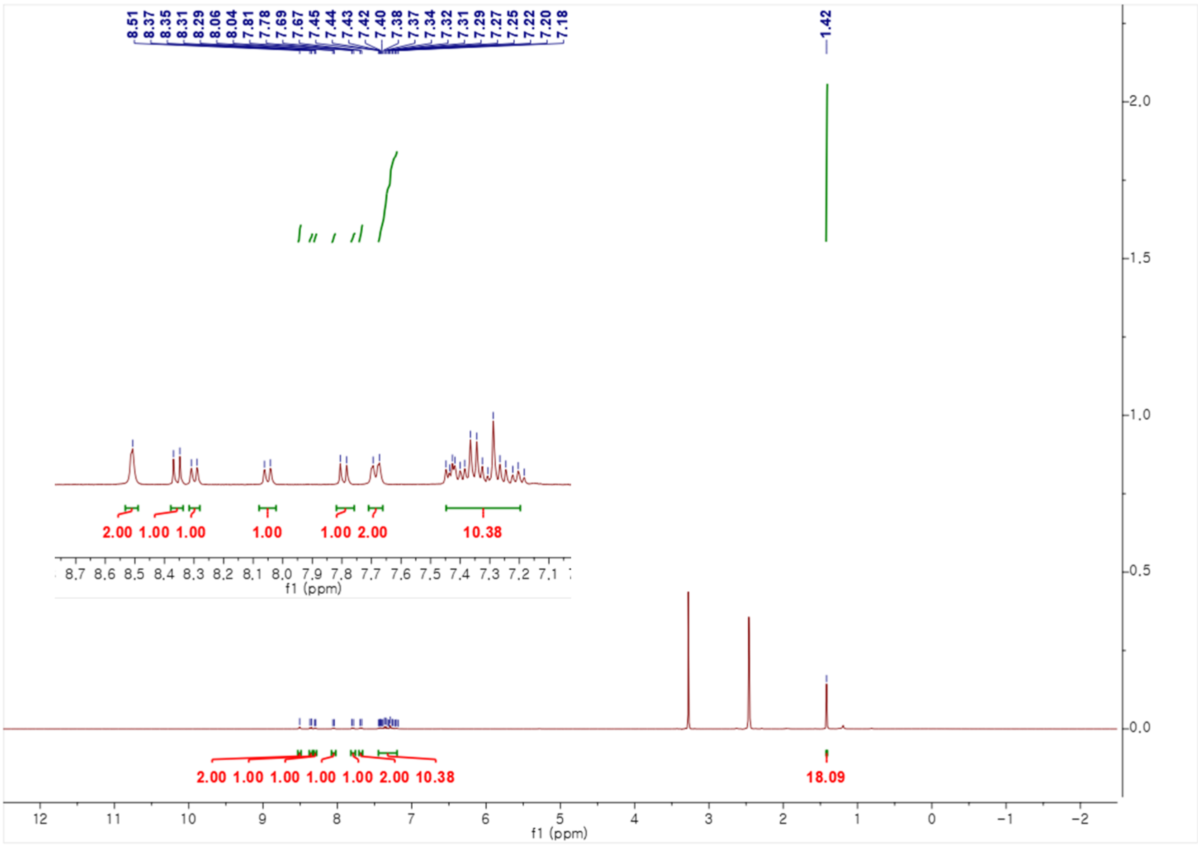

Fig. S10. ¹H NMR of **5-(2,12-di-tert-butyl-5,9-dioxa-13b-boranaphtho[3,2,1-de]anthracen-7-yl)-5H-benzo[b]carbazole (TDBA-Bz)**

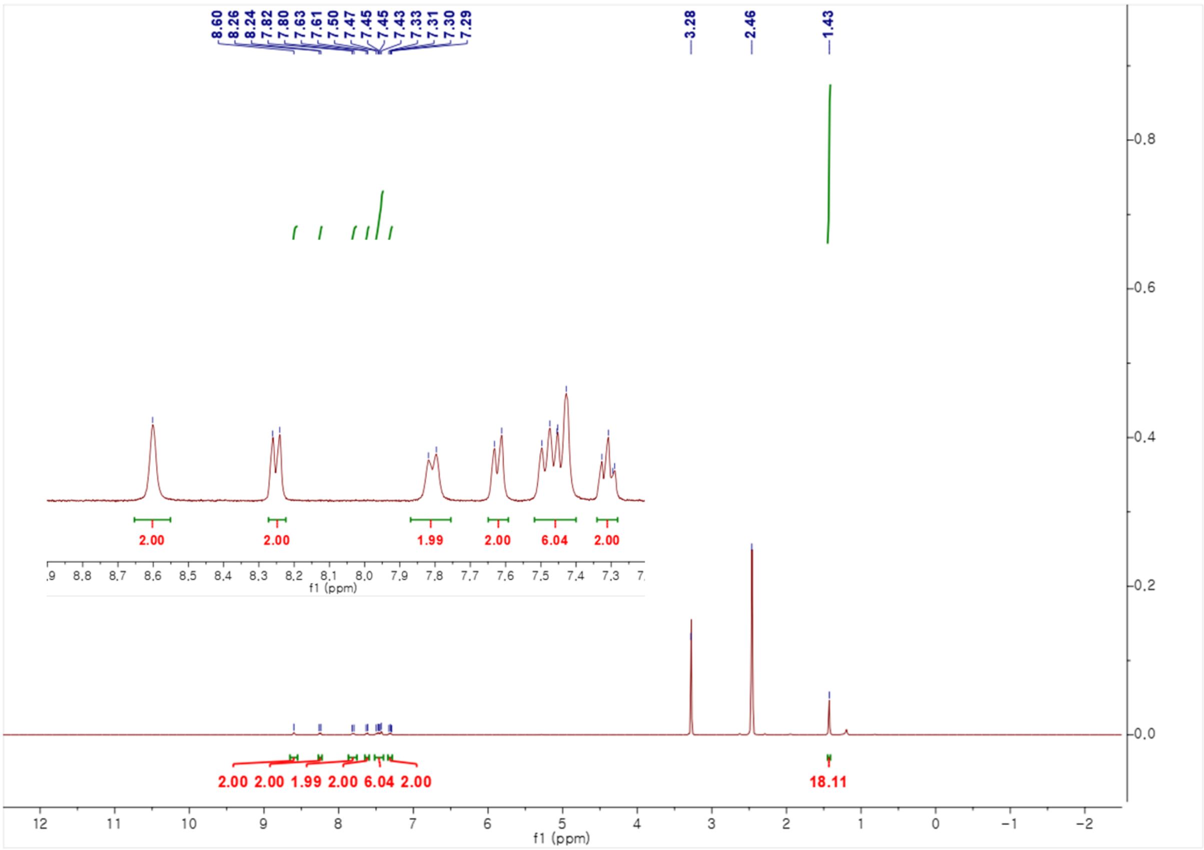

Fig. S11. ¹H NMR of **9-(2,12-di-tert-butyl-5,9-dioxa-13b-boranaphtho[3,2,1-de]anthracen-7-yl)-9H-carbazole (TDBA-Cz)**

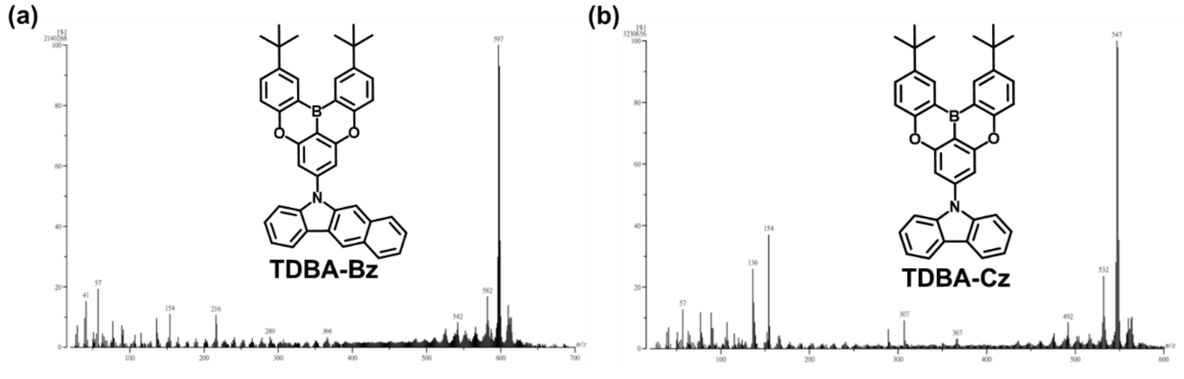

Fig. S12. HR-Mass spectroscopy: **(a) TDBA-Bz** and **(b) TDBA-Cz**
